# Joint Stress Analysis of the Navicular Bone of the Horse and Its Implications for Navicular Disease

**DOI:** 10.3390/bioengineering11010087

**Published:** 2024-01-17

**Authors:** Franz Konstantin Fuss

**Affiliations:** 1 Chair of Biomechanics, Faculty of Engineering Science, University of Bayreuth, D-95447 Bayreuth, Germany; franzkonstantin.fuss@uni-bayreuth.de; 2 Department of Biomechatronics, Division of Biomechanics, Fraunhofer Institute of Production Engineering and Automation IPA, D-95447 Bayreuth, Germany

**Keywords:** horse, navicular bone, navicular disease, joint force, joint stress, stress distribution, joint surface pressure, stress pole, centre of pressure

## Abstract

The horse’s navicular bone is located inside the hoof between the deep flexor tendon (DDFT) and the middle and end phalanges. The aim of this study was to calculate the stress distribution across the articular surface of the navicular bone and to investigate how morphological variations of the navicular bone affect the joint forces and stress distribution. Joint forces normalised to the DDFT force were calculated from force and moment equilibria from morphological parameters determined on mediolateral radiographs. The stress distribution on the articular surface was determined from the moment equilibrium of the stress vectors around the centre of pressure. The ratio of the proximal to the distal moment arms of the DDFT, as well as the proximo-distal position and extent of the navicular bone, individually or in combination, have a decisive influence on the position and magnitude of the joint force and the stress distribution. If the moment arms are equal and the bone is more proximal, the joint force vector originates from the centre of the joint surface and the joint load is evenly distributed. However, in a more distal position with a longer distal moment arm, the joint force is close to the distal edge, where the joint stress reaches its peak. Degenerative navicular disease, which causes lameness and pathological changes in the distal portion of the bone in sport horses, is likely to be more severe in horses with wedge-shaped navicular bones than in horses with square bones.

## 1. Introduction

**Figure 1:**
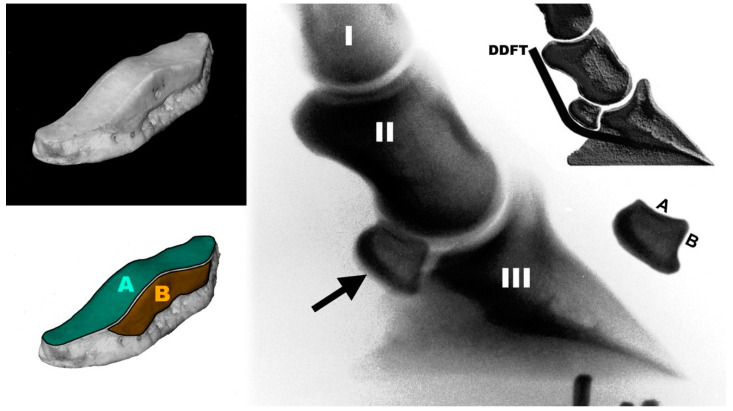
Navicular bone, iso-view, and medio-lateral radiograph (A: joint surface for the middle phalanx, B: joint surface for the hoof bone; I: proximal phalanx, II: middle phalanx, III: hoof (coffin) bone; arrow: navicular bone; DDFT: deep digital flexor tendon).

Unlike typical sesamoid bones, which are incorporated into a tendon or serve as an attachment for two tendinous shanks and are in contact with only one bone, the navicular bone (Figure 1) of the horse’s hoof is *not* included in a tendon and furthermore articulates with *two* bones. Therefore, it has two joint surfaces, one (A in Figure 1; naviculo-medio-phalangeal joint, NMP joint; Table 1) for the middle phalanx (short pastern bone) and the other (B in Figure 1; naviculo-disto-phalangeal joint, NDP joint) for the hoof or coffin bone (distal phalanx). The deep digital flexor tendon (DDFT) is wrapped around the navicular bone and, thus, exerts compressive forces rather than tensile forces on the navicular bone. The only similarity to a true sesamoid bone is that the navicular bone increases the moment arm of the tendon involved (DDFT). The navicular bone provides a constant insertion angle of the DDFT, and maintains its mechanical advantage [1,2] and also serves as an anticussion device [3,4,5].

The navicular bone is affected by degenerative navicular disease or podotrochleosis, which is one of the most common causes of performance-limiting lameness [6]. Navicular disease is a common syndrome in sport horses such as gallopers, jumpers, and Western horses, particularly in quarter horses [7]. Navicular disease is not only an overuse syndrome but also an inherited disease [8,9]. Vascular pathological changes occur mainly in the distal part of the bone: the arterial supply shifts from distal to proximal with increasing severity of the navicular disease [10,11], and the conical nutritional foramina transform into circular or mushroom-shaped canals [12]. Bentley et al. [13] found that navicular disease is associated with “high microcrack surface density” and “low bone volume fraction”. The navicular bone shows clear morphological variations that are also hereditary. Ueltschi et al. [9] differentiated three groups of specific navicular bone types (square, wedge-shaped, and trapezoid) in mediolateral radiographs of three groups of foals descended from three different stallions. Dik and van den Broek [14] associated severe degrees of navicular disease with the shape of the navicular bone in dorsopalmar radiographs, specifically when the proximal articular margin is convex. Since pathological changes can be easily diagnosed on lateral and dorsopalmar radiographs, a radiological assessment of the hooves is an integral element of pre-purchase examinations [15], an essential part of the horse purchasing process. Wilson et al. [16] calculated the force exerted by the DDFT on the navicular bone in sound horses and horses with navicular disease and concluded that this force was twice as large in the diseased cohort as in the control group, particularly in the early stance phase. The reason for this result was that the centre of pressure (origin of the ground reaction force) on the sole of the hoof was more cranial in the diseased group and was, therefore, responsible for a longer moment arm and a larger moment of the ground reaction force.

The literature gap to be addressed and filled in this study is that the polymorphic nature of the navicular bone [9] has, to date, never been considered for biomechanical studies. By filling this gap, a contribution to the literature is presented, relating to how different shapes of the navicular bone influence its loading pattern and stress distribution. Another contribution to the literature is the development of an analytical method for calculating the stress distribution, taking into account the fact that extreme loading cases (near an edge) could offload portions of an articular surface. The related research question is whether an apparent difference in the shape of the navicular bone could make a significant difference in the stress distribution in the sense that an unfavourable stress distribution could trigger or worsen the navicular disease.

The aim of this study is, therefore, to analyse the loading of the navicular bone (forces and joint surface stress), to relate morphological parameters to navicular bone mechanics, and to identify mechanically advantageous parameters. In addition, this study aims to provide a method for calculating forces and articular surface stresses acting on the navicular bone from lateral radiographs.

## 2. Materials and Methods

(A) Radiographs

The data for the biomechanical analysis were obtained from lateromedial forelimb radiographs (Figure 1) of 116 horses. A total of 98 radiographs were taken during pre-purchase examination. The remaining 18 were taken from cadaver samples, mounted on a rig with the hoof sole flat on the ground and under DDFT tension, with the DDFT marked with a thin steel wire [17].

(B) Biomechanical principles

**Figure 2:**
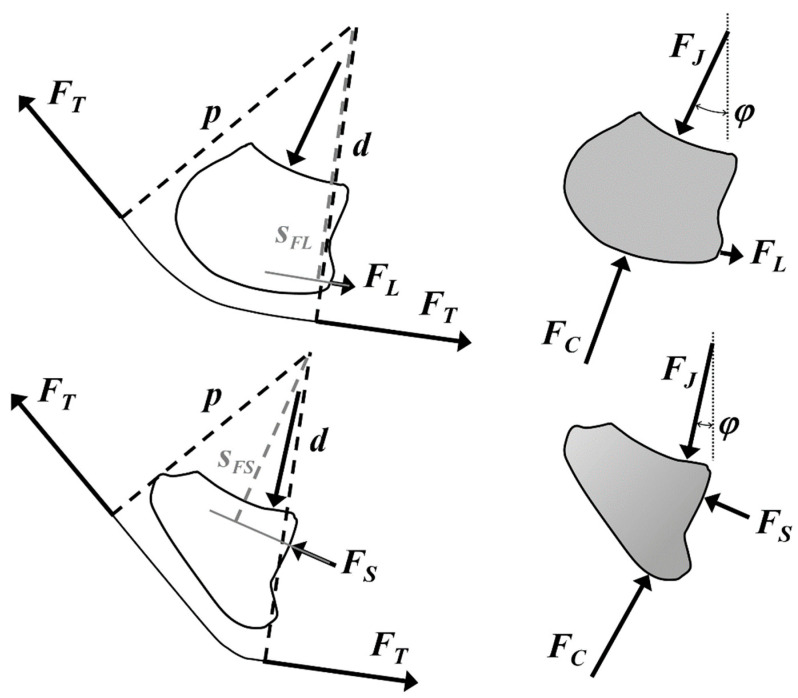
FBD and moment arms (proximal *p* and distal *d*) of the navicular bone; top row: square-shaped navicular bone with *p* ≥ *d* (requires *F_L_* for moment equilibrium); bottom row: wedge-shaped navicular bone with *p* < *d* (requires *F_S_* for moment equilibrium); the symbols are explained in Table 1.

**Figure 3:**
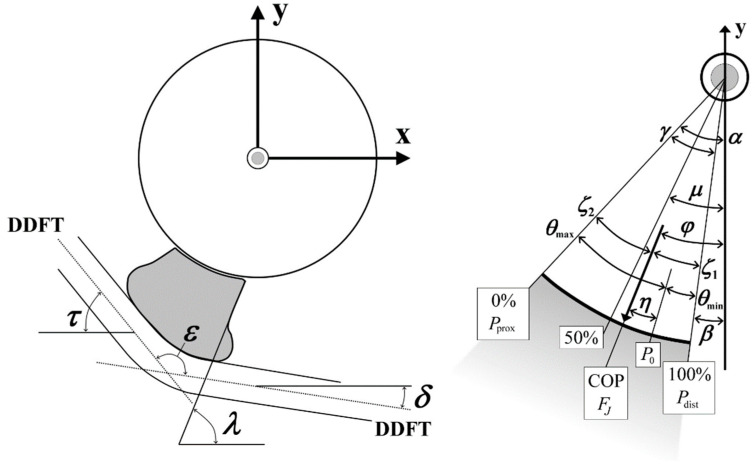
Coordinate system and angles of the navicular bone; “0%” = proximal border of the joint surface, “50%” = midpoint, “100%” = distal border; the *x*-axis is parallel to the sole surface of the hoof and points forward (cranial direction), the *y*-axis points upward (proximal); the symbols are explained in Table 1.

The centre of curvature (which was also used as the rotation centre in this study) of the coffin joint (distal interphalangeal joint) was determined by fitting a circle into the joint surface on the radiograph through three points (proximal and distal edges of the navicular joint surface, and cranial edge of the distal interphalangeal joint surface). With respect to the rotation centre, the moment arms (Figure 2) of the acting forces were measured considering the DDFT diameter (the force vectors of the tendon were placed in the centreline of the tendon). To make the navicular bone mechanics independent of the proximal tendon angle *τ* (Figure 3), *τ* was set at 50° with respect to the sole surface of the hoof because the pastern angle of the forelimbs is between 48° and 55° [18].

The proximal moment arm *p* of the DDFT force and that of the force of the navicular–hoof bone joint (B in Figure 1), *s_FS_*, or of the distal impar ligament, *s_FL_*, were normalised to the distal moment arm *d* of the DDFT force. The angles measured to define the geometry and position of the navicular bone are shown in Figure 3 and detailed in Table 1.

The free body diagram (Figure 2) used in this analysis consisted of the navicular bone as well as the DDFT section in contact with the navicular bone and all forces acting on them. These forces are (1) distal and (2) proximal force vectors *F_T_* of the DDFT; (3) the force of the joint between the navicular bone and the hoof bone, *F_S_*; or the distal impar ligament, *F_L_*, depending on which of the two is loaded; and (4) the joint force between the middle phalanx and navicular bone, *F_J_*. It was assumed that the friction on the lubricated articular surfaces and on the navicular bursa (between bone and DDFT) was negligible and, thus, the distal and proximal *F_T_* are equal.

The lateral collateral sesamoid ligament, which originates from the distal end of the proximal phalanx and is attached to the lateral angle of the navicular bone, was not included in the FBD because it relaxes during weight bearing, particularly when standing or in midstance when moving [19,20].

(C) Mathematical analysis

The biomechanical parameters calculated in this study were as follows:

(a) *F_S_* or *F_L_*, whichever is required for the moment equilibrium, normalised to *F_T_*;
(b) *F_J_*, normalised to *F_T_*;
(c) The direction of *F_J_* in terms of the angle *φ*;
(d) The position of *F_J_* at the articular surface (centre of pressure, COP; Figure 3);
(e) The articular surface pressure *P*.

(1) *Moment equilibrium about the rotation centre of the hoof joint*

The moment equilibrium (sum Σ of all moments *Mz* acting about the *z*-axis of the coordinate system) about the rotation centre of the hoof joint was calculated as follows:

(1) $$\Sigma Mz\;if\;d>p_{50}:\;-s_{FS}{\cdot F}_{S}+d{\cdot F}_{T}-p_{50}{\;\cdot F}_{T}+F_{J}\cdot 0=0\;\Sigma Mz\;if\;d=p_{50}:\;\;\;\;\;\;\;\;\;\;\;\;\;\;\;+d{\cdot F}_{T}-p_{50}{\;\cdot F}_{T}+F_{J}\cdot 0=0\;\Sigma Mz\;if\;d<p_{50}:\;+s_{FL}{\cdot F}_{L}+d{\cdot F}_{T}-p_{50}{\;\cdot F}_{T}+F_{J}\cdot 0=0$$

The moment arm *s_FS_* is shorter than *s_FL_*; however, only one of the two forces, *F_S_* or *F_L_*, is required for the moment equilibrium if *d* ≠ *p*_50_.

(2) *Force equilibrium*:

The force equilibrium (sum Σ of all forces *Fx* or *Fy* acting along the *x*- and *y*-axes of the coordinate system; Figure 3) was calculated as follows:

(2) $$\Sigma Fx\;if\;d>p_{50}:\;-F_{S}\sin\lambda_{S}+F_{T}\cos\delta -F_{T}\cos\tau +F_{Jx}=0\;\Sigma Fx\;if\;d=p_{50}:\;\;\;\;\;\;\;\;\;\;\;\;\;\;\;\;\;\;\;\;+F_{T}\cos\delta -F_{T}\cos\tau +F_{Jx}=0\;\Sigma Fx\;if\;d<p_{50}:\;+F_{L}\sin\lambda_{L}+F_{T}\cos\delta -F_{T}\cos\tau +F_{Jx}=0$$

(3) $$\Sigma Fy\;if\;d>p_{50}:\;+F_{S}\cos\lambda_{S}-F_{T}\sin\delta +F_{T}\sin\tau +F_{Jy}=0\;\Sigma Fy\;if\;d=p_{50}:\;\;\;\;\;\;\;\;\;\;\;\;\;\;\;\;\;\;\;\;-F_{T}\sin\delta +F_{T}\sin\tau +F_{Jy}=0\;\Sigma Fy\;if\;d<p_{50}:\;-F_{L}\cos\lambda_{L}-F_{T}\sin\delta +F_{T}\sin\tau +F_{Jy}=0$$

(3) *Joint forces:*

*F_S_* and *F_L_* are calculated from Equation (1), and *F_Jx_* and *F_Jy_* are obtained from Equations (2) and (3). The resultant joint force *F_J_* is obtained from the following equation:

(4) $$F_{J}=\sqrt{{F_{Jx}}^{2}+{F_{Jy}}^{2}}$$

(4) *Direction of the joint force and position of the centre of pressure (COP):*

The direction of *F_J_*, angle *φ* (Figure 2 and Figure 3; Table 1), located in the 3rd quadrant of the coordinate system (Figure 3), is expressed as the angle between negative *y*-axis and *F_J_*:

(5) $$\varphi =-\tan^{-1}\left(\frac{F_{Jx}}{F_{Jy}}\right)$$

The COP is obtained from

(6) $$\mathrm{COP}(\%)=100\frac{\varphi -\alpha }{\beta -\alpha }=100\frac{\varphi -\alpha }{\gamma }$$

where 0% and 100% correspond to the proximal and distal edges of the joint surface, respectively (Figure 3).

(5) *Calculation of the pressure distribution on the articular surface:*

In lubricated cylindrical joint surfaces covered with hyaline cartilage, the function of the distribution of pressure *P* on the joint surface is

(7) $$P_{\theta }=P_{0}\cos\theta$$

where *θ* is the angle between any point on the joint surface and the stress pole, and P_0_ denotes the maximal pressure at the stress pole where *θ* = 0 [21].

Due to lubrication, only normal forces act on the surface, so the frictional forces that cause shear stress can be assumed to be negligible. The normal forces distributed across the articular surface cause compressive contact stress *P_θ_*. To calculate *P_θ_* from Equation (7), we need to determine *P*_0_.

From first principles, the following equalities apply:

(a) The sum of *P_θ_* (times unit area) is equal to *F_J_*; more specifically, the sum of *P_θx′_* (times unit area), the *P_θ_*-component perpendicular to *F_J_*, is equal to 0 (force equilibrium), and the sum of *P_θy′_* (times unit area), the *P_θ_*-component parallel to *F_J_*, is equal to *F_J_*;
(b) The sum of *P_θ_*-moments about the COP is equal to 0 (moment equilibrium); the moment arm *l* of *P_θ_* is the shortest distance between *P_θ_* and the COP.

Before determining *P*_0_, we calculate the angle *η*, the angle between the **F_J_-** and **P_0_**-vectors. This is achieved by equating the integrals of *P_θx′_* and *P_θ_ l* to 0 (force and moment equilibriums), as shown subsequently.

*Force equilibrium:*

The boundaries of the weight-bearing area are the distal and proximal edges of the articular surface, denoted by *θ_dist_* and *θ_prox_*, respectively (Figure 3). Note that *θ_dist_* − *θ_prox_* = *γ*. However, if surface extends more than 0.5π on the distal or proximal side of the stress pole, *θ_dist_* or *θ_prox_* are set to −0.5π or +0.5π, respectively, since *P_θ_* cannot be negative. Negative pressure means that the articular surface were under tensile stress when the articular surfaces were not in loose contact.

(8) $$P_{\theta x}=P_{\theta }\sin\theta =P_{0}\cos\theta \sin\theta$$

(9) $$P_{\theta y}=P_{\theta }\cos\theta =P_{0}\cos^{2}\theta$$

The x- and y-components of the surface stress must be calculated in terms of the joint force *F_J_* and not in terms of *P*_0_. The position of the force vector *F_J_* on the articular surface is defined as the centre of pressure (COP). If the articular surface angles on both sides of the stress pole are unequal, i.e., *θ_prox_* + *θ_dist_* ≠ 0, then the COP, i.e., the origin of *F_J_*, does not coincide with the stress pole, i.e., the origin of *P*_0_. Thus, the load is distributed asymmetrically. The angle between the force vector **F_J_** and the maximum pressure vector **P_0_** is denoted by *η*. At *P*_0_, *θ* = 0; at *F_J_*, *θ* + *η* = 0. The angles with respect to the COP and *F_J_* are denoted by *ζ*, where

(10) ζ=θ+η

Thus, the x’- and y’-components of the surface stress with respect to the joint force *F_J_* are

(11) $$P_{\theta x’}=P_{\theta }\sin\zeta =P_{\theta }\sin(\theta +\eta )=P_{0}\cos\theta \sin(\theta +\eta )$$

(12) $$P_{\theta y’}=P_{\theta }\cos\zeta =P_{\theta }\cos(\theta +\eta )=P_{0}\cos\theta \cos(\theta +\eta )$$

Considering that the x’- and y’-components of the surface stress are aligned with the joint force *F_J_*, so that *F_J_* points downward, in the negative y’-direction, integration of Equations (11) and (12) across the joint surface area returns zero and *F_J_*, respectively. For reasons of comparison, the articular surface is simplified as a cylindrical surface with constant radius *R* and mediolateral width *W*. When integrating over *θ*, from *θ_prox_* to *θ_dist_*, *W* and *R* are set to unity to normalise the stress values.

(13) $$W\;R\;P_{0}\int_{\zeta_{2}-\eta }^{\zeta_{1}-\eta }\cos\theta \cos(\theta +\eta )\;d\theta =F_{J}$$

(14) $$W\;R\;P_{0}\int_{\zeta_{2}-\eta }^{\zeta_{1}-\eta }cos\theta \sin(\theta +\eta )\;d\theta =0$$

where

(15) $$\zeta_{1}-\eta =\theta_{dist}\left(\leq +\pi /2\right)$$

(16) $$\zeta_{2}-\eta =\theta_{prox}\left(\geq -\pi /2\right)$$

*θ_dist_* and *θ_prox_*, with respect to the stress pole, are calculated from two angles on either side of the COP and *F_J_*, namely from *ζ*_1_ and *ζ*_2_ (Figure 3):

(17) $$\zeta_{1}=\beta -\varphi$$

(18) $$\zeta_{2}=\alpha -\varphi =\zeta_{1}-\gamma$$

*Moment equilibrium:*

Calculating *η* from the moment equilibrium about the COP depends on the basic definition of the COP: all surface pressure vectors (times unit area) are in equilibrium about the COP. The moment is equal to *P_θ_* times unit area multiplied by the shortest distance between *P_θ_* and the COP. The latter distance is the moment arm *l*, which is a function of *θ*:

(19) l=Rsin⁡(θ+η)

In Equation (19), we must again consider that *l* is calculated in terms of the COP and not in terms of *P*_0_, and thus, in terms of *ζ*. The moment arm *l* must be zero at the COP, i.e., at *η* = −*θ*, and not at the stress pole where *θ* = 0.

Substituting and integrating over *θ* gives the overall moment *M_z_* about the *z*-axis, which must be zero.

(20) $$W\;R^{2}\;P_{0}\int_{\zeta_{2}-\eta }^{\zeta_{1}-\eta }\cos\theta \sin(\theta +\eta )\;d\theta =0$$

Equations (14) and (20) must and expectedly yield the same integral to reduce to

(21) $$\int_{\zeta_{2}-\eta }^{\zeta_{1}-\eta }cos\theta \sin(\theta +\eta )\;d\theta =0$$

*Calculation of η if the entire articular surface is loaded:*

Solving Equation (21) for *η* by simplifying and applying summation laws yields:

(22) $$\eta =\tan^{-1}\frac{\cos\left(2\zeta_{1}\right)-\cos\left(2\zeta_{2}\right)}{\sin\left(2\zeta_{2}\right)-\sin\left(2\zeta_{1}\right)+2\left(\zeta_{1}-\zeta_{2}\right)}=\tan^{-1}\frac{\cos\left(2\zeta_{1}\right)-\cos\left(2\zeta_{2}\right)}{\sin\left(2\zeta_{2}\right)-\sin\left(2\zeta_{1}\right)+2\gamma }\;$$

If *ζ*_1_ + *ζ*_2_ = 0, i.e., the COP is at 50%, and then *η* = 0, and thus, *P*_0_ originates from the COP.

Once *η* is known, *P*_0_ is calculated from Equation (13)

(23) $$P_{0}=\frac{4F_{J}}{W\;R}\;\;\frac{1}{\sin\eta \left(\cos2\zeta_{2}-\cos2\zeta_{1}\right)+\cos\eta \left(\sin2\zeta_{1}-\sin2\zeta_{2}+2\zeta_{1}-2\zeta_{2}\right)}$$

Equation (23) defines the unique relationship between the joint force vector **F_J_** originating from the COP and the peak joint stress vector **P_0_** originating from the stress pole. *η* defines the angle between these two vectors. *η* is independent of the magnitude of the vectors and depends only on the relative position of the COP within the articular surface, defined by angles *ζ*_1_ and *ζ*_2_. From *η* calculated from Equation (22), we obtain *θ_prox_* and *θ_dist_* from Equations (15) and (16).

*Calculation of η if the joint surface is partially loaded:*

If *θ_prox_* < −π/2 (or *θ_dist_* > +π/2), then any stress at |*θ*|> π/2 would be tensile if the mating articular surfaces were not in loose contact. Therefore, *γ* must be adjusted and limited to the area that is effectively subjected to compressive stress, and particularly limited to *γ*_eff_. This is achieved by reducing *θ*_prox_ to −π/2 (or *θ_dist_* to +π/2), with the stress equal to zero. Consequently, *η* changes to *η*_eff_.

If *θ_prox_* < −π/2:

(24) $$\theta_{prox\_eff}=\zeta_{2\_eff}-\eta_{eff}=-\pi /2$$

(25) $$\theta_{dist\_eff}=\zeta_{1}-\eta_{eff}$$

As a result, we obtain two unknowns, namely *η*_eff_ and *θ_dist__*_eff_. However, relative to the *ζ*-angles, the two unknowns are *η*_eff_ and *ζ*_2_eff_.

Substituting

(26) $$\zeta_{2\_eff}=\eta_{eff}-\frac{\pi }{2}$$

into Equation (22) yields

(27) $$\frac{\cos\left(2\zeta_{1}\right)-\cos\left(2\eta_{eff}-\pi \right)}{\sin\left(2\eta_{eff}-\pi \right)-\sin\left(2\zeta_{1}\right)+\left(2\zeta_{1}-2\eta_{eff}+\pi \right)}-\tan\eta_{eff}=0$$

Solving Equation (27) numerically delivers the unknown variable *η*_eff_ (<π/2) and, subsequently, from Equations (24) and (25), *θ_pro_*_x_eff_ and *θ_dist_*__eff_.

Alternatively, *η*_eff_ (<π/2) is obtained directly from a non-linear regression function *f*, where *η*_eff_ = *f* (*ζ*_1_):

For 0° ≤ *ζ*_1_ ≤ 90°, *η*_eff_ (in degrees) is

*η*_eff_ = 90 − 2.02∙*ζ*_1_ + 0.0026∙*ζ*_1_^2^ + 9.62×10^−5^∙*ζ*_1_^3^ + 4.13×10^−6^∙*ζ*_1_^4^ − 9.79×10^−8^∙*ζ*_1_^5^ + 8.16×10^−10^∙*ζ*_1_^6^ − 3.02×10^−12^∙*ζ*_1_^7^ + 4.20×10^−15^∙*ζ*_1_^8^ (28)

For 5° ≤ *ζ*_1_ ≤ 35° (range of the current dataset, although only data of *ζ*_1_ < 15° are relevant), *η*_eff_ (in degrees) is

*η*_eff_ = 90 − 1.99∙*ζ*_1_ − 0.00093∙*ζ*_1_^2^ + 0.0003∙*ζ*_1_^3^ − 2.02×10^−6^∙*ζ*_1_^4^ (29)

For small *ζ*_1_, the fit functions of Equations (28) and (29) reduce to 90 − 2 *ζ*_1_. As *ζ*_1_ → 0°, *η*_eff_ → 90°, but *η*_eff_ is mathematically not defined at *ζ*_1_ ≡ 0° since the first term of Equation (27) is reduced to 0/0.

Finally, from *η*_eff_, *ζ*_2*_*eff_, *θ_dist__*_eff_, and *θ_prox__*_eff_, the adjusted joint stress parameters, *P*_0_eff_, *P_dist_*__eff_, and *P_prox_*__eff_, are recalculated from Equations (23) and (7).

In rare, if not theoretical cases, if *θ_dist_* > +π/2 (maximum *θ_dist_* in the current dataset: 81°):

(30) $$\theta_{prox\_eff}=\zeta_{2}-\eta_{eff}$$

(31) $$\theta_{dist\_eff}=\zeta_{1\_eff}-\eta_{eff}=+\pi /2$$

As a result, we obtain two unknowns, namely *η*_eff_ and *θ*_prox*_*eff_. However, relative to the *ζ*-angles, the two unknowns are *η*_eff_ and *ζ*_1_eff_.

Substituting

(32) $$\zeta_{1\_eff}=\eta_{eff}+\frac{\pi }{2}$$

into Equation (22) yields

(33) $$\frac{\cos\left(2\eta_{eff}+\pi \right)-\cos\left(2\zeta_{2}\right)}{\sin\left(2\zeta_{2}\right)-\sin\left(2\eta_{eff}+\pi \right)+\left(2\eta_{eff}+\pi -2\zeta_{2}\right)}-\tan\eta_{eff}=0$$

Solving Equation (33) numerically delivers the unknown variable *η*_eff_ (>−π/2) and, subsequently, from Equations (30) and (31), *θ_pro_*_x_eff_ and *θ_dist_*__eff_.

Finally, from *η*_eff_, *ζ*_2*_*eff_, *θ_dist__*_eff_, and *θ_prox__*_eff_, the adjusted joint stress parameters, *P*_0_eff_, *P_dist_*__eff_, and *P_prox_*__eff_, are recalculated from Equations (23) and (7).

(D) Regression analysis

To assess how the morphological parameters influence the biomechanical parameters, multiple regression was applied to specific datasets.

When multiple regression is used to identify the unique (individual) and shared (combined) influence of two predictors (independent variables) on the response variable (dependent variable) rather than isolating the most influential predictor, it is necessary to determine whether multiple regression is warranted. This justification was rejected based on at least one of the following two criteria:

(a) Negative shared component (B, squared semi-partial correlation coefficient; if B < 0, then there is no shared component [22]);
(b) Variance inflation factor (VIF) greater than 5 [23]; VIF = 1/(1 − r_mult_^2^); r_mult_^2^ = A + B + C.

If a multiple regression was justified, the unique (A, C) and the shared (B) variances were calculated from

B = r_1sing_^2^ + r_2sing_^2^ − r_mult_^2^ (34)

A = r_1sing_^2^ − B (35)

C = r_2sing_^2^ − B (36)

where r_sing_^2^ and r_mult_^2^ are the coefficients of determination of single or multiple regressions, respectively.

## 3. Results

(A) Morphological parameters

The position and extent of the navicular bone below the head of the middle phalanx (Figure 1) are defined by the angles *α* (proximal edge) and *β* (distal edge). The position angle *μ* of the navicular bone indicates whether the navicular bone is more proximal or distal in relation to the head of the middle phalanx, while the extension angle *γ* refers to the included angle of the articular surface (NMP joint, A in Figure 1) in relation to long or short navicular bones in the proximo-distal direction. The statistical details of the morphological parameters are listed in Table 2.

**Figure 4:**
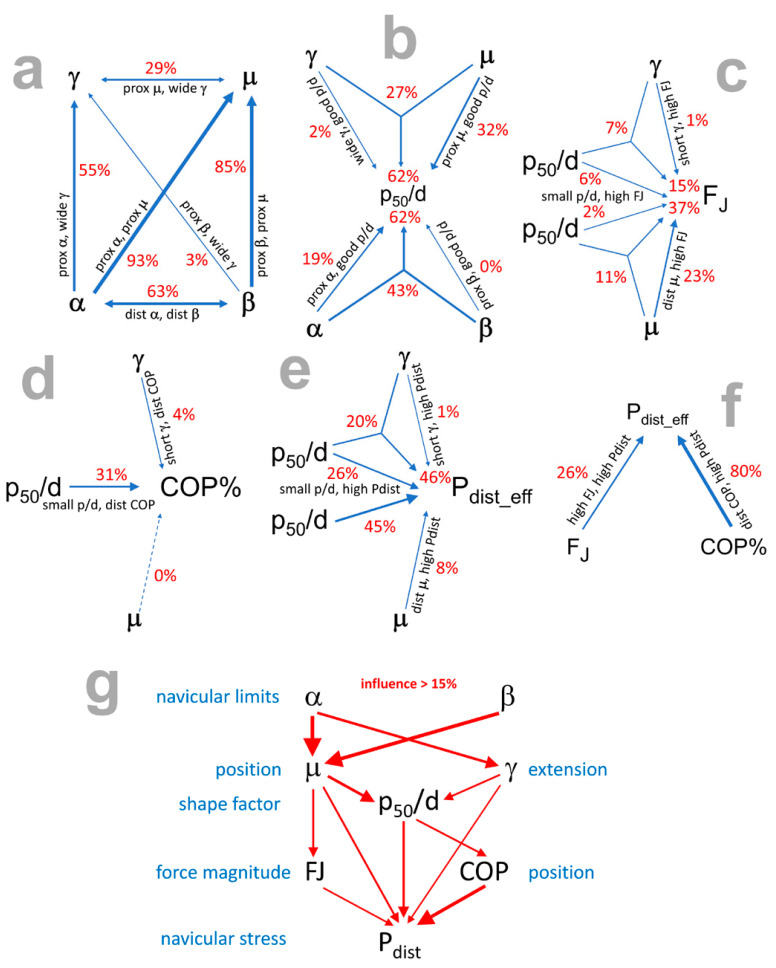
Influences between morphological and biomechanical parameters; the symbols are explained in Table 1; prox = proximal; dist = distal; the width of the arrows corresponds to the strength of the influence; (**a**)interrelation of *α*, *β*, *γ* and *μ*; (**b**) influence of *α*, *β*, *γ* and *μ* on *p*_50_/*d*; (**c**) influence of *γ*, *μ* and *p*_50_/*d* on *F_J_*; (**d**) influence of *γ*, *μ* and *p*_50_/*d* on COP%*;* (**e**) influence of *γ*, *μ* and *p*_50_/*d* on *P*_dist_eff_; (**f**) influence of COP% and *F_J_* on *P*_dist_eff_; (**g**) summary of influences >15%.

Since *γ* and *μ* are calculated directly from *α* and *β*, i.e., *γ* = *β* − *α* and *μ* = (*α* + *β*)/2, the correlation of *α* and *β* with *γ* or *μ* leads to a multiple regression r^2^ of one, which means that a multiple regression is not justified (VIF = ∞). Single regressions (Figure 4a) of *α* and *β* with *γ* show that *α* influences *γ* in 55% (r^2^ = 0.5501, p < 0.0001), while *β* influences *γ* in only 3% (r^2^ = 0.0327, p = 0.0266). The reason for this result is that the range of *β* is smaller than that of *α*. Conversely, *α* and *β* show a comparable influence on *μ* in 93% (r^2^ = 0.9325, p < 0.0001) and 85% (r^2^ = 0.8548, p < 0.0001), respectively.

The proximal moment arm of the DDFT, *p*_50_/*d*, normalised to the distal one, explains the shape of the navicular bone. Long *p*_50_/*d* (≈1) occur in rectangular navicular bones, while short *p*_50_/*d* (≈0.8) occur in wedge-shaped navicular bones. The overall influence of *α* and *β* and *γ* and *μ* is 62% (multiple regression r^2^ = 0.6172; Figure 4b and Table 3). Notably, the unique influences of *γ* and *β* on *p*_50_/*d* are very small, 2.2% and 0.3%, respectively (Figure 4b and Table 3).

(B) Biomechanical parameters.

The biomechanics of the navicular bone is characterised by the following variables:

(1) The normalised magnitude of the joint forces *F_J_*, *F_S_*, and *F_L_*;
(2) The position of the COP.

Both parameters determine the articular surface stress, again characterised by the following variables:

(3) The normalised magnitude of the peak stress vector;
(4) The stress distribution (even or uneven).

The navicular bone is primarily loaded on two opposite sides:

- At the NMP joint (A in Figure 1), by the force *F_J_* (Figure 2);
- At its underside, where the navicular bone is in contact with the deflected DDFT by the force *F_C_* (Figure 2).

Therefore, the navicular bone is compressed by these two forces. Furthermore, the navicular bone experiences forces on its distal side:

- If *p*_50_/*d* > 1, then the distal impar ligament (tensile force *F_L_*) is under tension;
- If *p*_50_/*d* < 1, then it is loaded with pronounced compressive force *F_S_* at the NDP joint (B in Figure 1).

The forces *F_L_* and *F_S_* amount to a maximum (worst case) of 6.4% and 36% of *F_J_*, respectively. The force ratios of *F_L_*/*F_J_* and *F_S_*/*F_J_* correlate well with *p*_50_/*d* (r^2^ = 0.9505), with a regression equation of *F_L_*/*F_J_* ∨ *F_S_*/*F_J_* ≈ 2 *p*_50_/*d* − 2.

The statistical details of all biomechanical parameters are listed in Table 4.

The influence of the morphological parameters, particularly *γ*, *μ*, and *p*_50_/*d*, on the biomechanical parameters is explained as follows:

(a) Joint force *F_J_*:

The influence of *γ* and *p*_50_/*d* on *F_J_* was 15% (multiple regression r^2^ = 0.1465, p = 0.0001). The unique influences of *γ* and *p*_50_/*d* and the shared (squared semi-partial correlation) influence were 1.4%, 5.9%, and 7.4%, respectively (Figure 4c and Table 3). The influence of *μ* and *p*_50_/*d* on *F_J_* was 37% (multiple regression r^2^ = 0.3672, p < 0.0001). The unique influences of *μ* and *p*_50_/*d* and the shared influence were 23.5%, 2.0%, and 11.2%, respectively (Figure 4c, Table 3). The strongest morphological influence on *F_J_* came from the angle *μ*.

(b) COP:

The influence of *γ* and *p*_50_/*d* on the COP was 32% (multiple regression r^2^ = 0.3232, p < 0.0001). The single regressions (squared partial correlations of the multiple regression) showed a difference in their coefficients of determination: *γ* with COP by a small r^2^ = 0.0395 (p = 0.0167) and *p*_50_/*d* with COP by a larger r^2^ = 0.3083 (p < 0.0001). The relatively small r^2^ of *γ* with COP (although still significant) led to small, unique influences of *γ* and *p*_50_/*d* and small, shared influence of 1.5%, 28.4%, and 2.5%, respectively (Figure 4d and Table 3). In this case, a multiple regression does not provide any more information than the single regressions.

The influence of *μ* and *p*_50_/*d* on the COP showed a strong influence of 79% (multiple regression r^2^ = 0.7865, p < 0.0001). The single regressions showed a striking discrepancy in their coefficients of determination: *μ* with COP by r^2^ = 0.0001 (p = 0.4439) and *p*_50_/*d* with COP by r^2^ = 0.3083 (p < 0.0001). This result raises the question of how single influences of 0% and 31% result in a multiple influence of 79%. The answer is readily apparent when consulting the unique influences of *μ* and *p*_50_/*d* and their shared influence of 48% (0.4782), 79% (0.7864), and −48% (−0.4781), respectively (Figure 4d and Table 3). The negative B-value indicates that there is no shared component. Compared to the previous example, where the unique and shared influences of *γ* were small, the result of this example is that unique and shared influences of *μ* were significant, of approximately +50% and −50%. However, due to their different signs, they cancel each other out. The single regression r^2^ of 0.0001, statistically insignificant with p = 0.4439, excludes multiple regression from the outset.

Of the three morphological parameters, *γ*, *μ*, and *p*_50_/*d*, the latter has the only serious influence on the COP (location of the COP within the joint surface) with 31%. The two angles, *γ* and *μ*, have no direct influence on the COP but rather an indirect influence via the *p*_50_/*d*, influencing the length of the moment arm *p*_50_/*d* with 62%.

(c) Stress at the distal edge of the navicular bone (*P*_dist_eff_):

The smaller the *p*_50_/*d*, the more the COP and, thus, the joint force vector *F_J_* shift towards the distal edge of the navicular joint surface, and the larger is *F_S_* (Figure 2). When *p*_50_/*d* = 1 or *p*_50_/*d* = 0.8, the mean relative stress *P*_dist_eff_ at the distal border is 1 or 3.75, respectively.

The influence of *γ* and *p*_50_/*d* on *P*_dist_eff_ was 46% (multiple regression r^2^ = 0.4628, p < 0.0001). The corresponding unique influences of *γ* and *p*_50_/*d* and the shared influence were 1.2%, 25.5%, and 19.6%, respectively (Figure 4e and Table 3).

The influence of *μ* and *p*_50_/*d* on *P*_dist_eff_ was 59% (multiple regression r^2^ = 0.5880, p < 0.0001). The corresponding unique influences of *μ* and *p*_50_/*d* and the shared influence were 13.7%, 50.8%, and −5.7%, respectively (Figure 4e and Table 3). The fact that the shared influence is negative rules out multiple regression. The single regressions are interpreted as follows: *μ* with *P*_dist_eff_ by r^2^ = 0.0799 (p = 0.0011), and *p*_50_/*d* with *P*_dist_eff_ by r^2^ = 0.4511 (p < 0.0001). However, the single regression of *γ* with COP by r^2^ = 0.0395 (p = 0.0167) had a smaller r^2^ (12.8% of the other single regression r^2^) than *μ* with *P*_dist_eff_ by r^2^ = 0.0799 (17.7% of 0.4511). The strongest morphological influence on *P*_dist_eff_ came from the moment arm *p*_50_/*d* with 45%.

In addition to the influence of morphological parameters, the influence of *F_J_* and COP on *P*_dist_eff_ can also be examined. The influence of both biomechanical parameters on *P*_dist_eff_ was 93% (multiple regression r^2^ = 0.9270, p < 0.0001). This means that VIF = 13.7, i.e., VIF > 5, which excludes a multiple regression. The single regression r^2^ of *F_J_* and COP with *P*_dist_eff_ were 0.2574 (p < 0.0001) and 0.8030 (p < 0.0001), respectively (Figure 4f and Table 3). As expected, the location of the COP within the joint surface has a stronger influence on the magnitude of *P*_dist_eff_.

The influences between morphological and biomechanical parameters are summarised in Figure 4g. If one excludes weak influences < 15%, *F_J_* is only influenced by *μ*, and COP by *p*_50_/*d* (and indirectly by *μ* via *p*_50_/*d*). Missing strong influences are *β* on *γ*, *γ* on COP (only via *p*_50_/*d*), *p*_50_/*d* on *F_J_*, *μ* on COP (only via *p*_50_/*d*), and *γ* on *F_J_*.

**Figure 5:**
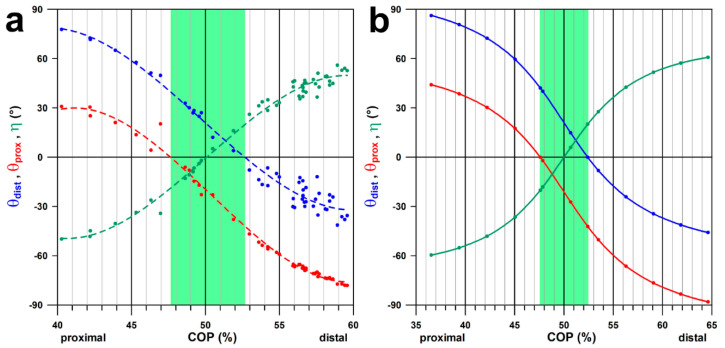
*θ*_dist_, *θ*_prox_, and *η* against COP (%); (**a**): calculated data from radiographs; (**b**): simulated data; the green area corresponds to the position of *P*_0_ within the joint surface.

The stress pole, where the stress *P*_0_ originates, should not be confused with the COP, the origin of *F_J_*. While the COP is always located at the joint surface, *P*_0_ can move outside the joint surface and then become a virtual stress pole. In fact, *P*_0_ is located inside the joint surface only if the COP lies within a small window of 50% ± 2–3% (determined empirically, based on the processed data; Figure 5a), i.e., when *θ*_dist_ is positive and *θ*_prox_ is negative. *P*_0_ is outside the joint surface if both *θ* angles share the same sign, be it negative or positive. This means that *P*_0_ becomes virtual and, thus, is no longer relevant if it lies outside the articular surface. Consequently, *P*_dist_ and *P*_prox_ must be calculated to determine the peak pressure. We can, therefore, define three conditions for stress distributions:

- *P*_0_ within the articular surface: peak pressure at *P*_0_, where *θ*_dist_ is positive, and *θ*_prox_ is negative;
- *P*_0_ outside the articular surface on proximal side: peak pressure at *P*_prox_, where both *θ*_dist_ and *θ*_prox_ are positive;
- *P*_0_ outside the articular surface on distal side: peak pressure at *P*_dist_, where both *θ*_dist_ and *θ*_prox_ are negative.

If *P*_0_ is at the proximal edge of the joint surface, then *θ*_prox_ = 0 and *ζ*_2_ = *η*. If *P*_0_ is at the distal edge of the joint surface, then *θ*_dist_ = 0 and *ζ*_1_ = *η*.

When simulating an average navicular bone with average values of *α*, *β*, *λ_S_*, *δ*, *s_FS_*/*d* (Table 1), but with *p*_50_/*d* between 0.999 and 0.9, so that 34% < COP < 66% (regression equation: COP% ≈ −10/3 *p*_50_/*d* + 11/3), *P*_0_ is within the joint surface only when the COP is at 50% ± 2.45% (Figure 5b). The reason why *p*_50_/*d* was varied as opposed to the other constant parameters was that the COP was most strongly correlated with *p*_50_/*d* (Table 3).

**Figure 6:**
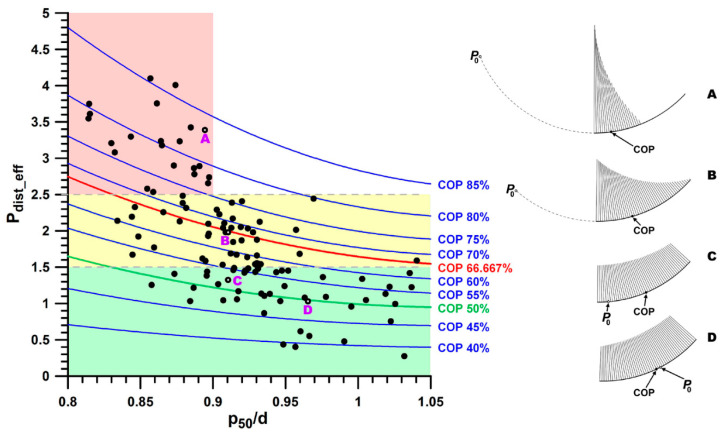
Distal stress *P*_dist_eff_ versus *p*_50_/*d*; the isolines of the COP positions are indicated on the graph; four different stress distributions (A, B, C, D) are shown on the right side of the figure, corresponding to four data points on the graph; the 3 coloured zones (green, yellow, red) indicate acceptable, increased, and excessive stress, respectively.

If the joint force vector *F_J_* is not exactly at COP = 50%, i.e., it does not correspond to *P*_0_, an asymmetrical load and stress distribution occurs on the joint surface (Figure 6). The position of the COP correlates well with the relative distal stress *P*_dist_eff_ at r^2^ = 0.8030 (Table 3). When COP is 50%, *P*_dist_eff_ is about 1. When COP is 80%, the relative distal stress *P*_dist_eff_ is about 3.75. In addition, with a COP > 66.67%, the proximal part of the joint surface is no longer required for loading (Figure 6, case A). The smaller the distal stress *P*_dist_, the larger *γ* and *μ* (larger surface angle and more proximal position; Table 3 and Table 5). The more proximal the navicular bone (angle *μ*), the larger the included angle *γ* and the moment arm ratio *p*_50_/*d*, the smaller the joint forces and stresses, and the more uniform the stress distribution (Figure 6, Table 5).

Therefore, the mechanical ‘design’ strategy (Table 5) to avoid adverse loading of the navicular bone is as follows:

(1) Increase *p*_50_;
(2) Increase *γ*;
(3) Both strategies 1 and 2 imply a reduction of *μ* (since both *p*_50_/*d* and *γ* are negatively correlated with *μ*) and, thereby, rotate the navicular bone in the proximal direction.

## 4. Discussion

The results of this study impressively show that variations in joint morphology have an influence on joint mechanics, especially on articular stress distribution.

**Figure 7:**
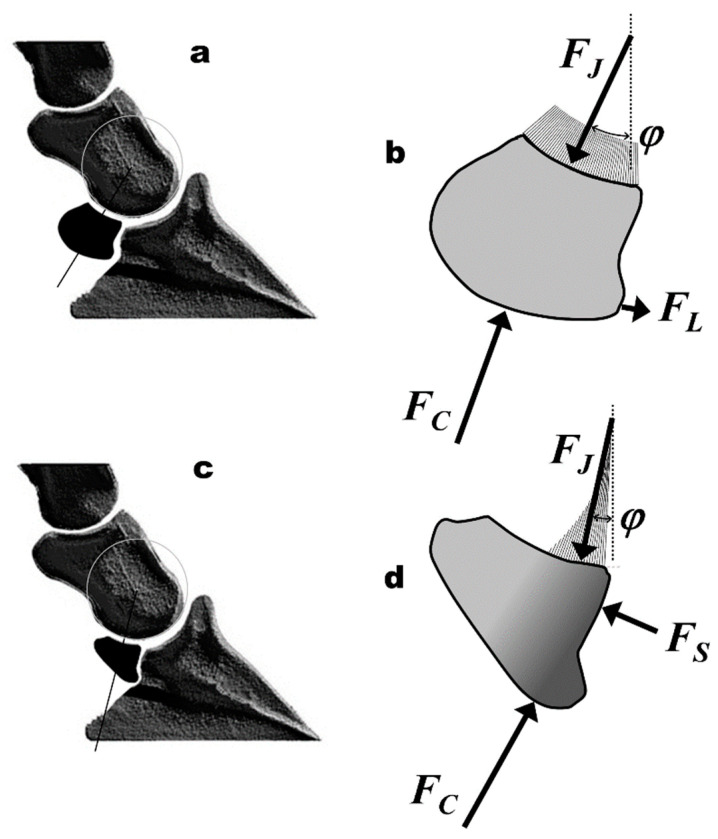
Navicular bones with different shapes ((**a**,**b**): square-shaped; (**c**,**d**): wedge-shaped) at different positions (angle *μ*, (**a**): proximal position, (**c**): distal position), and the corresponding free-body diagrams with stress distributions at the joint surface (NMP, naviculo-mediophalangeal joint).

The aim of this study was to identify mechanically ideal and unfavourable morphological parameters. The strength of this study is that it provides scientific evidence that the shape of the navicular bone has a critical influence on its loading pattern and stress distribution. Furthermore, this study provides an analytical method for calculating the stress distribution, even for cases where portions of an articular surface are unloaded, and the extent of the unloaded portion is unknown. The most important morphological influencing factors appear to be the position of the navicular bone (angle *μ*; Table 5 and Figure 7) and the included articular surface angle *γ*, as they influence the moment arm ratio *p*_50_/*d* (Figure 4). All three morphological parameters show a direct effect on the biomechanical parameters, namely the magnitude of the joint forces, the position of the COP, the magnitude of the peak pressure, and the pressure distribution. The adverse mechanical parameters are large, normalised joint forces and peak pressures, eccentric COP, and uneven pressure distribution (Table 5, Figure 7). The negative effect of uneven stress distribution becomes evident from Figure 6. Figure 6 (case D) represents uniform pressure distribution (the stress vectors are almost the same size with a slight central peak). Figure 6 (case A) shows extremely uneven loading (only the distal half of the articular surface is loaded). The latter loading case leads to an excessive stress peak at the distal edge of the articular surface and, thus, to overloading of this region. Such high stress is mechanically detrimental to both cartilage and bone.

The practical application of the method and the results described in this article is that the method is useful as an additional diagnostic tool when measuring the morphological parameters directly on the radiograph and applying the equations described in the Methods section. The results of this method can be conveniently included in any pre-purchase examination and can also be applied to the selection of breeding stock since the shape of the navicular bone appears to be hereditary [9]. The most important mechanical parameters to consider are the moment arm ratio *p*_50_/*d* of the DDFT and the position of the COP, which is highly correlated with the relative distal pressure *P*_dist_. The COP should not be >67% (Figure 6), and an almost uniform pressure distribution can be seen when COP = 50 ± 3% (Figure 5).

The new findings from the present study are that the morphology of the navicular bone has a direct influence on its loading. A distally overloaded navicular bone is likely to be the trigger for navicular disease, as pathological changes also occur in the distal sector, namely abnormal fluid in the medullary cavity [24], a shift of the arterial supply from distal to proximal under increasing degrees of navicular disease [10,11] and shape changes to the distal nutritional foramina [12,25]. However, further research is needed to confirm such a hypothesis. The knowledge about morphological variations and their biomechanical implications appears even more important as breeding selection can prevent the hereditary transmission of unfavourable navicular bone morphology. The inheritance of navicular disease could be due to the fact that morphology is hereditary [9], which, in turn, affects the joint force and bone stress distribution.

**Figure 8:**
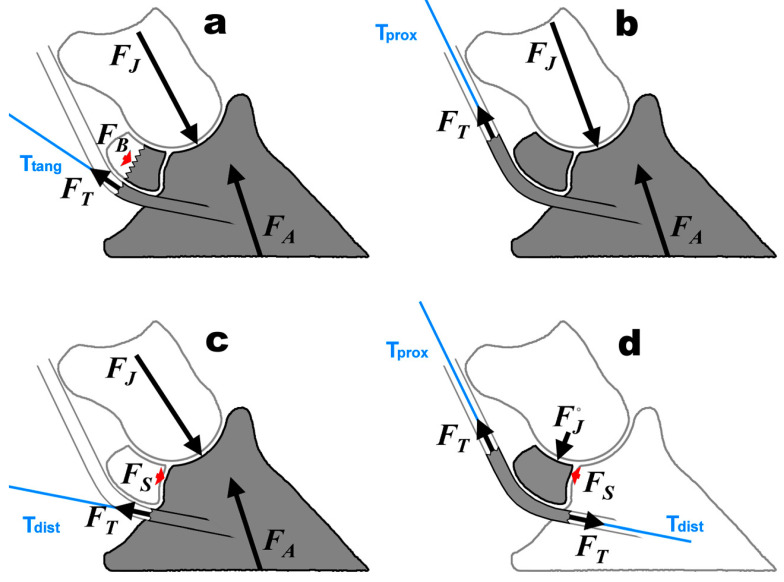
Different free body diagrams; shaded bones are inside the FBD, contoured ones are outside; *F_A_* = ground reaction force (between foot strike and mid-stance), *F_B_* = bone stress inside the bisected navicular bone; note that the joint forces *F_J_* in all 4 subfigures are not of the same magnitude and depend on the nature and corresponding force equilibrium of the FBD; (**a**): FBD when defining the line of action (T_tang_) of the DDFT as the tangent to the tendon at the point where the moment arm intersects the curved DDFT, wrapped around the flexor surface at the palmar border, (**b**): hoof + navicula (r bone (T_prox_: line of action of the DDFT), (**c**): isolated hoof bone (T_dist_: line of action of the DDFT), (**d**): isolated navicular bone.

Willemen et al. [26] and Wilson et al. [16] examined and calculated the joint force of the navicular bone. Wilson et al. [16] concluded that the compressive force exerted by the DDFT on the navicular bone is higher in the first 70% of the stance phase in horses with navicular disease. The methods of Willemen et al. [26] and Wilson et al. [16] are not sufficiently mechanically accurate because the free-body diagram (FBD) was not correctly isolated and because not ***all*** forces acting on the FBD were considered. It is a common flaw in FBDs involving the navicular bone that the moment arm of the DDFT is drawn from the rotation centre to the “*palmar border*” [16,26] of the navicular bone (Figure 8a). The line of action of the DDFT is, thus, defined as the tangent to the tendon at the point where the moment arm intersects the deflected tendon and wraps around the flexion surface at the palmar margin (Figure 8a). However, the correct action line of a tendon, muscle, or ligament is usually constructed at the boundary, where the FBD is separated from, or “cut out” of, the reference frame, which is the external world. Thus, the preferred moment arm *p* results from bisecting the DDFT on the proximal side of the navicular bone (Figure 8b) rather than halving the navicular bone itself (Figure 8a). The latter method must take into account bone stress (Figure 8a) acting on the distal half of the navicular bone, which is unknown in the first place and results in a four-force member FBD. Alternatively, the FBD of Figure 8a could be drawn without bisecting the navicular bone, but then the force exerted by the DDFT on the proximal half of the navicular bone must be taken into account since the DDFT was bisected at the centre of its curvature. As another alternative, the DDFT could be cut on the distal side of the navicular bone (thereby excluding the navicular bone from the FBD; Figure 8c), again resulting in a four-force member FBD, because the cut occurs at the level of the NDP joint. In contrast to that, the FBD of Figure 8b is a three-force member and allows the calculation of the *F_T_* when the ground reaction force is known. Subsequently, *F_S_* or *F_L_* is determined from the four-force member FBD of Figure 8d.

The nomenclature terms for two joints between the navicular bone on the one hand and the middle and distal phalanges on the other hand are not specified in the ‘*Illustrated Veterinary Anatomical Nomenclature*’ [27]. The reason for this is that these two joints are only small parts of the distal interphalangeal joint (DIP joint) with no medical significance (in contrast to the navicular bone itself). However, they have a biomechanical significance, as both joints carry and transmit loads. Therefore, the two joints need to be named anatomically. In analogy to the metacarpophalangeal joint (MCP joint), the joints between the navicular bone (os naviculare) and the distal phalanx (phalanx distalis) or the middle phalanx (phalanx media) should be referred to as the naviculo-distophalangeal joint (NDP joint, Figure 1) or the naviculo-mediophalangeal joint (NMP joint, Figure 1), respectively, as already mentioned in the Introduction. The terms *distophalangeal* and *proximophalangeal* are nevertheless found in the literature. Duffy et al. [28] used the term “distophalangeal joints” for the DIP joints. Yeung et al. [29] used the term “proximophalangeal joints” for the proximal interphalangeal (PIP) joints. Owen [30], on the other hand, used the term proximophalangeal as a synonym for metacarpal (…*the two metacarpal or proximo-phalangeal bones* … *extend forward*…) in Archeopteryx skeletons.

The limitations of this study are threefold:

(1) The stress distribution across the articular surface was not modeled based on Hertzian stress because the joint surfaces are composed of hyaline cartilage, characterised by low elastic modulus and viscoelastic properties. In addition, a clearance between the corresponding joint surfaces, i.e., the difference in radii of curvature, was not considered either due to the above-mentioned properties and due to the lubrication with synovia, a viscous fluid.
(2) There is no conclusive evidence available in the literature that increased stress on the navicular bone is the primary cause of navicular disease. There is some circumstantial evidence based on clinical studies. Wilson et al. [16] found that the force exerted on the navicular bone by the DDFT was twice as large in the diseased cohort as in the control group. The reason for this finding was unspecified heel pain that forced the pressure centre on the sole of the hoof into a cranial position to relieve the pain. The cranial position of the centre of pressure, in turn, increased the moment arm of the ground reaction force at the coffin joint and, therefore, also the force of the DDFT, thereby compressing the navicular bone more than usual. Analgesia of the palmar digital nerves reversed this mechanism, and the calculated force acting on the navicular bone decreased [31]. However, the cause of this pathobiomechanical mechanism of unloading the heel coupled with overloading of the navicular bone cannot logically and conclusively lie in a painful navicular disease. Accordingly, McGuigan and Wilson [31] correctly state that “*this mechanism identifies navicular disease as a **possible** end point for a variety of heel related conditions.*” However, the most important conclusion related to this mechanism is that in two horses with similarly overloaded navicular bones, as a result of relief from heel pain, the horse with a more wedge-shaped navicular bone is likely to experience greater stress on the articular surfaces and inside the navicular bone. Bentley et al. [13] found that navicular disease is associated with “high microcrack surface density”. Due to these circumstances, this study can only suggest that there is a higher risk of navicular disease if *P*_dist_eff_ is large, specifically in navicular bones with adverse morphology. This study, in turn, represents an appropriate method to initiate an expanded study of the cause of navicular disease by examining horses diagnosed with navicular disease based on radiological signs and/or significant lameness. The proposed method outlined in this study is independent of actual ground reaction forces (which are obviously smaller in the lame limb) since the forces of the model are normalised to the DDFT force. Caution is advised when it comes to the training load on a horse, as frequent overloading of the navicular bone, e.g., in gallopers or trotters, can theoretically lead to disease in the navicular bone despite ideal morphological conditions.
(3) The multiple regressions calculated to examine the influence of morphological parameters on biomechanical parameters were performed with two predictors, even if the number of morphological parameters was three (*γ*, *μ,* and *p*_50_/*d*). Multiple regression with three predictors would be the method of choice, although the above-mentioned problems with negative shared variance with three predictors would be more complex, making interpretation difficult.

## 5. Conclusions

This research study sheds new light on the biomechanics of the navicular bone and offers a new aspect of it. The fact that the navicular bone has different shapes when viewed from the side [9] and that these shapes were apparently inherited from the horses’ parents (at least confirmed by stallion data [9]) is already known from the literature. It was not previously known from the literature that the shape of the navicular bone has a significant influence on the stress distribution on its articular surface. Regardless of other factors that lead to navicular disease, the shape of the navicular bone alone could be the deciding factor as to whether a horse is more or less susceptible to developing navicular disease.

## Figures and Tables

**Table 1:** Notation, abbreviations, and symbols.

Symbol(s)	Explanation
	***Abbreviations***
NMP	naviculo-mediophalangeal joint
NDP	naviculo-distophalangeal
DDFT	deep digital flexor tendon
COP	centre of pressure (origin of joint forces or ground reaction forces); position of the COP on the joint surface of the navicular bone: 0% at the proximal border, 100% at the distal border
FBD	free body diagram
r, r^2^	coefficients of correlation and determination
p	probability (p-value)
A, B, C	components of multiple regression (B = squared semi-partial correlation; A + B and B + C = squared partial correlations)
*R*	radius
*W*	width
	***Coordinate system***
x	forward (cranial direction), parallel to the sole of the hoof
y	upward (proximal direction), perpendicular to the sole of the hoof
	***Angles of the navicular bone measured with respect to the coordinate syste*** **m**
*α*	angle between the negative *y*-axis and the articular surface radius at the proximal border of the joint surface (*α* is negative)
*β*	angle between the negative *y*-axis and the articular surface radius at the distal border (negative angle when *β* opens on the proximal side of the *y*-axis)
*γ*	included angle of the joint surface (between proximal and distal border); *γ = β − α*
*μ*	position angle of the navicular bone; angle between the negative *y*-axis and the articular surface radius at the midpoint of the joint surface; *μ* = (*α* + *β*)/2 (*μ* is negative)
*τ*	angle between the negative *x*-axis and the DDFT proximal to the navicular bone
*δ*	angle between the positive *x*-axis and the DDFT distal to the navicular bone (negative when DDFT pointing upward towards phalanx III)
*ε*	included angle of the DDFT
*λ_L_*	angle between *x*-axis and a line perpendicular to the distal impar sesamoid ligament
*λ_S_*	angle between *x*-axis and the joint between navicular and hoof bone
	***Forces and pressure***
*F_J_*	main joint force (acting from the middle phalanx towards the navicular bone; naviculo-mediophalangeal joint)
*φ*	angle between the negative *y*-axis and the joint force *F_J_* (*φ* is negative because it lies on proximal side of *y*-axis)
*F_S_*	additional joint force from hoof bone to navicular bone (naviculo-distophalangeal joint)
*F_L_*	force of the distal impar ligament
*F_T_*	force of the DDFT
*F_C_*	compressive force of the navicular bone, resultant of distal and proximal *F_T_*
*P*	surface pressure on the navicular joint surface (contact stress between middle phalanx and the navicular bone)
*P*_0_	maximal stress at the stress pole
*P*_dist_	stress at the distal border of the navicular bone
*P*_prox_	stress at the proximal border
	***Moment arms with respect to the rotation centre of the hoof joint***
*p*	moment arm of the DDFT proximal to the navicular bone
*p*_50_	*p* at *τ* = 50°
*d*	moment arm of the DDFT distal to the navicular bone
*s_FS_*	moment arm of *F_S_*
*s_FL_*	moment arm of *F_L_*
	***Angles measured with respect to F_J_ and its COP***
*ζ*_1_	angle between *F_J_* and the articular surface radius at the distal border of the joint surface (*ζ*_1_ is positive, counter-clockwise)
*ζ*_2_	angle between *F_J_* and the articular surface radius at the proximal border (*ζ*_2_ is negative, clockwise)
*η*	angle between *F_J_* and *P*_0_
	***Angles measured with respect to P_0_***
*θ*	angle between the articular surface radius at any point on the joint surface and *P*_0_
*θ*_dist_	angle between *P*_0_ and the articular surface radius at the distal border
*θ*_prox_	angle between *P*_0_ and the articular surface radius at the proximal border

**Table 2:** Statistics of morphological parameters; the symbols are explained in Table 1 and Figure 3.

	Mean	Standard Deviation	Minimum	Maximum	Range
*p*_50_/*d*	0.916	0.051	0.814	1.040	0.226
*s_FS_*/*d*	0.590	0.049	0.458	0.771	0.313
*s_FL_*/*d*	0.877	0.017	0.851	0.898	0.047
*λ_S_* (°)	66	6.83	51	85.5	34.5
*λ_L_* (°)	59.85	5.79	51	71	20
*δ* (°)	4.11	5.54	−9	18	27
*α* (°)	−42.39	7.58	−63	−20.5	42.5
*β* (°)	−0.24	5.17	−16	10	26
*γ* (°)	42.15	4.69	29.5	58	28.5
*μ* (°)	−21.32	6.05	−38	−5.75	32.25

**Table 3:** Multiple regressions (MR); symbols of variables are detailed in Table 1; A, B, C: components of multiple regression (B = squared semi-partial correlation; A + B and B + C = squared partial correlations; A and C: unique or individual influence of predictors *a* and *c* on the response variable; B: shared or combined influence of predictors *a* and *c* on the response variable); r^2^: coefficient of determination; p: p-value (probability); VIF: variance inflation factor; UX: fraction of the response variable not explained from the multiple regression; C1 and C2: criteria for justifying rejection of multiple regression (cf. Section 2, correlation analysis).

	MR 1	MR 2	MR 3	MR 4	MR 5	MR 6	MR 7	MR 8	MR 9
predictor *a*	*α*	*γ*	*γ*	*μ*	*γ*	*μ*	*γ*	*μ*	*F_J_*
predictor *c*	*β*	*μ*	*p*_50_/*d*	*p*_50_/*d*	*p*_50_/*d*	*p*_50_/*d*	*p*_50_/*d*	*p*_50_/*d*	COP%
response variable	*p*_50_/*d*	*p*_50_/*d*	*F_J_*	*F_J_*	COP%	COP%	*P*_dist_eff_	*P*_dist_eff_	*P*_dist_eff_
A + B + C r^2^	0.6172	0.6172	0.1465	0.3672	0.3232	0.7865	0.4628	0.5880	0.9270
A + B + C p	<0.0001	<0.0001	<0.0001	<0.0001	<0.0001	<0.0001	<0.0001	<0.0001	<0.0001
B + C r^2^	0.4313	0.5952	0.1326	0.1326	0.3083	0.3083	0.4511	0.4511	0.8030
B + C p	<0.0001	<0.0001	<0.0001	<0.0001	<0.0001	<0.0001	<0.0001	<0.0001	<0.0001
A + B r^2^	0.6139	0.2946	0.0879	0.3470	0.0395	0.0001	0.2077	0.0799	0.2574
A + B p	<0.0001	<0.0001	0.0007	<0.0001	0.0167	**0.4439**	<0.0001	0.0011	<0.0001
VIF	2.61	2.61	1.2	1.6	1.5	4.7	1.9	2.4	**13.7**
UX	0.3828	0.3828	0.8535	0.6328	0.6768	0.2135	0.5372	0.412	0.073
B	0.4280	0.2726	0.0740	0.1124	0.0246	**−0.4781**	0.1960	**−0.0570**	0.1334
A	0.1859	0.0220	0.0139	0.2346	0.0149	0.4782	0.0117	0.1369	0.1240
C	0.0033	0.3226	0.0586	0.0202	0.2837	0.7864	0.2551	0.5081	0.6696
trend 1	proximal *β*, long *p*_50_/*d*	proximal *μ*, long *p*_50_/*d*	short *p*_50_/*d*, high *F_J_*	short *p*_50_/*d*, high *F_J_*	short *p*_50_/*d*, distal COP	short *p*_50_/*d*, distal COP	short *p*_50_/*d*, high *P_dist_*	short *p*_50_/*d*, high *P_dist_*	distal COP, high *P_dist_*
trend 2	proximal *α*, long *p*_50_/*d*	wide *γ*, long *p*_50_/*d*	small *γ*, high *F_J_*	distal *μ*, high *F_J_*	small *γ*, distal COP	----	small *γ*, high *P_dist_*	distal *μ*, high *P_dist_*	high *F_J_*, high *P_dist_*
MR justified: *Y*/*N*	*Y*	*Y*	*Y*	*Y*	*Y*	*N*	*Y*	*N*	*N*
justifi-cation if *N*	---	---	---	---	---	C1	---	C1	C2

**Table 4:** Statistics of mechanical parameters; the symbols are explained in Table 1.

	Mean	Standard Deviation	Minimum	Maximum	Range
*F_J_*/*F_T_*	0.789	0.100	0.564	1.041	0.477
*F_S_*/*F_T_*	−0.162	0.071	−0.341	−0.008	0.333
*F_L_*/*F_T_*	0.029	0.014	0.003	0.046	0.043
*φ* (°)	−16.65	6.83	−33.58	−2.10	31.48
COP (%)	61.26	8.88	38.97	83.57	44.60
*η*_eff_ (°)	45.05	30.24	−50.51	74.67	125.18
*ζ*_1_ (°)	16.41	4.55	7.72	30.52	22.80
*ζ*_2___eff_ (°)	−23.87	3.91	−33.38	−13.85	19.53
*θ_dist_*__eff_ (°)	−28.65	34.01	−66.95	81.03	147.98
*θ_prox_*__eff_ (°)	−68.93	31.65	−90	31.03	121.03
*P*_0___eff_	2.94	2.03	0.98	10.01	9.03
*P_dis_*_t___eff_	1.87	0.83	0.27	4.10	3.83
*P_prox_*__eff_	0.41	0.44	0	1.94	1.94
*γ*_eff_ (°)	40.28	6.68	23.05	58	34.95

**Table 5:** Ideal and adverse loading cases (c.f. Figure 6 and Figure 7).

**Variables**	**Ideal Case Figure 7a,b)**	**Adverse Case (Figure 7c,d)**
***morphological variables***	***influencing the biomechanical parameters***	
included joint surface angle *γ* (proximodistal extent)	wide (>40°) (long)	small (<40°) (short)
proximodistal position *μ*	proximal (>20°)	distal (<20°)
shape factor *p*_50_/*d*	0.9–1 (rectangular or trapezoid)	<0.9 (cuneiform or wedged)
***biomechanical variables***	***influenced by the morphological parameters***	
navicular joint force (*F_J_*/*F_T_*)	small (<0.75)	large (>0.85)
force of navicular–hoof bone joint (*F_S_*/*F_T_*)	0	large (>0.2)
location of COP	50% (central)	>67% (distal)
pressure distribution	even	uneven (distal stress peak)
*P_dist_*__eff_ (Figure 6)	moderate (~1)	high (>2.5)
*P_prox_*__eff_ (Figure 6)	moderate (~1)	0

## Data Availability

The data presented in this study are available on request from the author to any qualified researcher.
